# Chinese herbal medicine and its active compounds in attenuating renal injury *via* regulating autophagy in diabetic kidney disease

**DOI:** 10.3389/fendo.2023.1142805

**Published:** 2023-03-03

**Authors:** Peng Liu, Wenhui Zhu, Yang Wang, Guijie Ma, Hailing Zhao, Ping Li

**Affiliations:** ^1^Shunyi Hospital, Beijing Hospital of Traditional Chinese Medicine, Beijing, China; ^2^Renal Division, Department of Medicine, Heilongjiang Academy of Chinese Medicine Sciences, Harbin, China; ^3^Beijing Key Lab for Immune-Mediated Inflammatory Diseases, China-Japan Friendship Hospital, Beijing, China

**Keywords:** diabetic kidney disease (DKD), autophagy, Chinese medicine, active compounds, herbal extracts

## Abstract

Diabetic kidney disease (DKD) is the main cause of end-stage renal disease worldwide, and there is a lack of effective treatment strategies. Autophagy is a highly conserved lysosomal degradation process that maintains homeostasis and energy balance by removing protein aggregates and damaged organelles. Increasing evidence suggests that dysregulated autophagy may contribute to glomerular and tubulointerstitial lesions in the kidney under diabetic conditions. Emerging studies have shown that Chinese herbal medicine and its active compounds may ameliorate diabetic kidney injury by regulating autophagy. In this review, we summarize that dysregulation or insufficiency of autophagy in renal cells, including podocytes, glomerular mesangial cells, and proximal tubular epithelial cells, is a key mechanism for the development of DKD, and focus on the protective effects of Chinese herbal medicine and its active compounds. Moreover, we systematically reviewed the mechanism of autophagy in DKD regulated by Chinese herb compound preparations, single herb and active compounds, so as to provide new drug candidates for clinical treatment of DKD. Finally, we also reviewed the candidate targets of Chinese herbal medicine regulating autophagy for DKD. Therefore, further research on Chinese herbal medicine with autophagy regulation and their targets is of great significance for the realization of new targeted therapies for DKD.

## 1 Introduction

Diabetic nephropathy (DKD), one of the most prevalent complications of diabetes mellitus, is the leading cause of end-stage kidney disease (1, 2). DKD accounts for 30% to 50% of all chronic kidney disease (CKD) cases, covering 285 million people worldwide (3). Because of its high prevalence, poor prognosis, and healthcare cost burden, DKD is a major public health problem. The development of DKD is quite complicated and involves chronic low-grade inflammation, accumulation of advanced glycosylation end products and oxidative stress. Despite some progress in the treatment of DKD, such as controlling blood glucose, lipids, blood pressure and lifestyle modification, there is still no specific treatment (4, 5).

**Figure 1:**
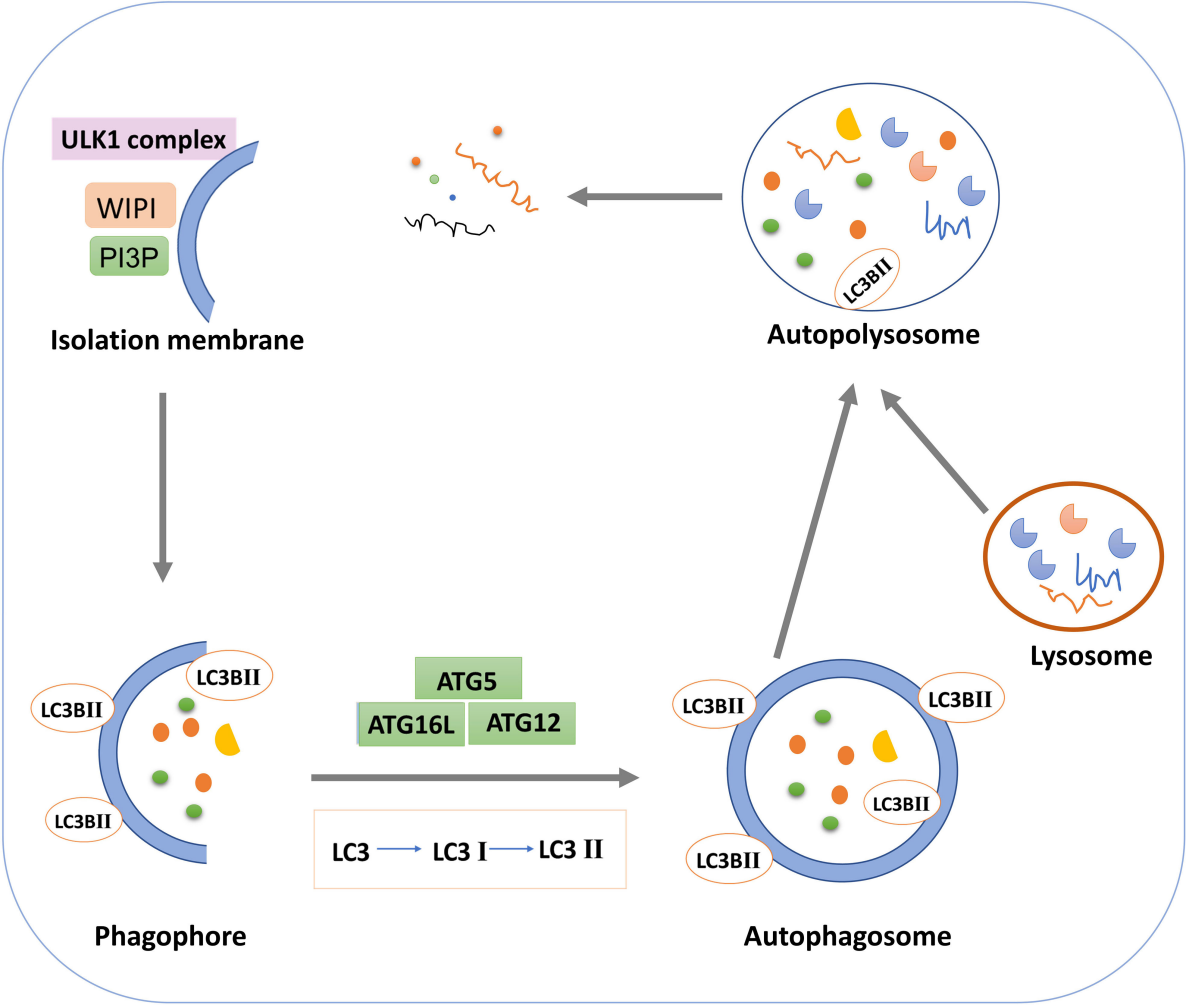
Schematic diagram of macroautophagy.

**Figure 2:**
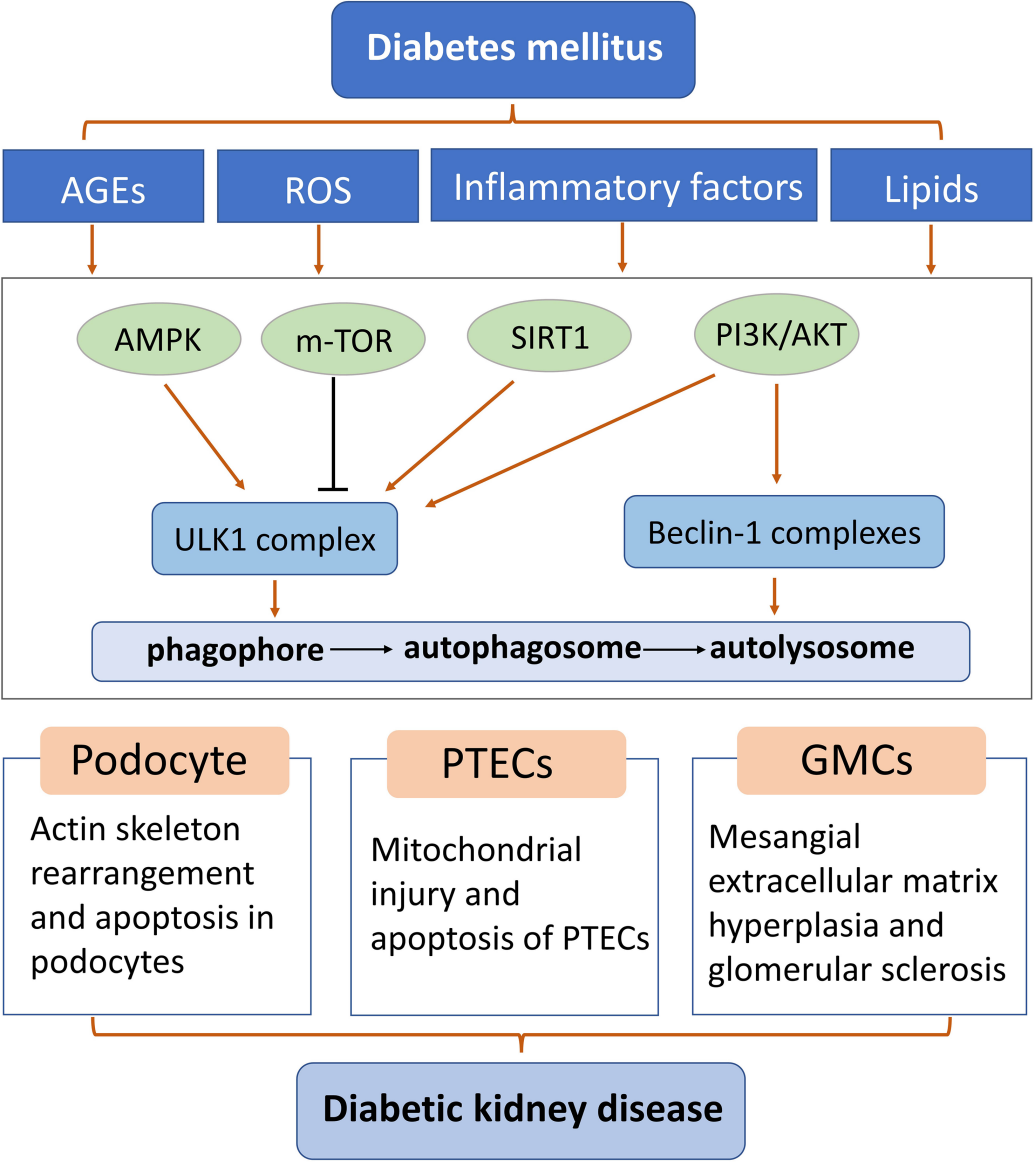
Diagram of some mechanisms of autophagy in podocytes, glomerular mesangial cells, and proximal renal tubular epithelial cells of diabetic kidney disease. AGEs: advanced glycation end products; GMCs: glomerular mesangial cells; PTEC: proximal renal tubular epithelial cells; ROS: reactive oxygen species.

**Figure 3:**
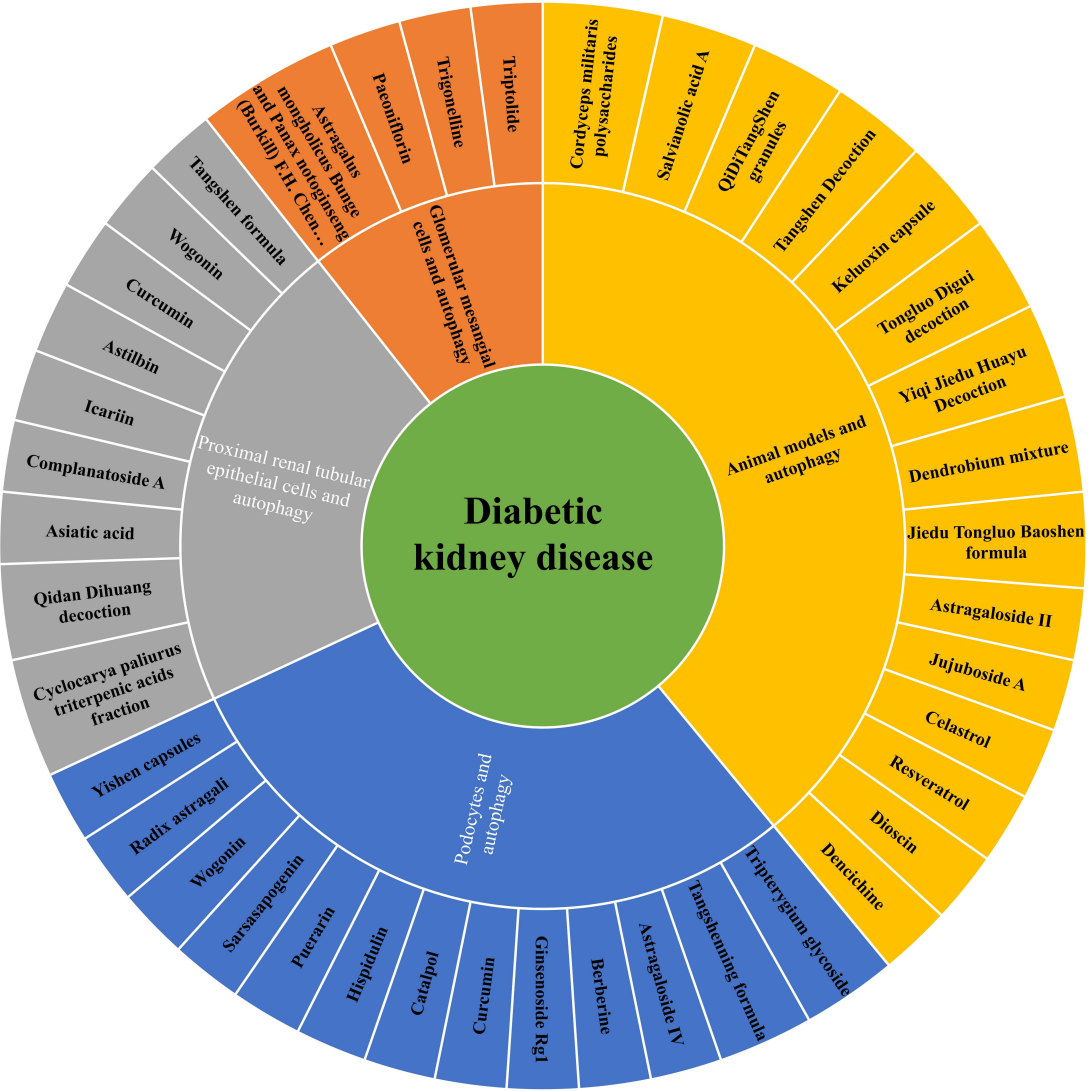
The role of Chinese herbal compound preparations, single herbs and active compounds against renal injuries *via* regulating autophagy in DKD.

To date, a growing number of studies have been conducted to determine the association of autophagy with the progression of DKD (6, 7). In this review, we outline recent advances in the study of autophagy in DKD, with a particular focus on the relationship between autophagy and podocytes, glomerular mesangial cells, and proximal renal tubular epithelial cells in **Figures 1**, **2**. Here, we present the role and mechanism of herbal medicines to improve the effect on DKD by regulating autophagy in **Tables 1**–**3** and **Figure 3**. In addition, herbal medicines with autophagy-modulating ability are highlighted as a promising therapeutic strategy for the treatment of DKD.

**Table 1:** The role of Chinese herbal compound preparations against renal injuries *via* regulating autophagy in DKD.

Signaling Pathways	Chinese herbal compound preparations	Herbal composition	*In Vivo/In Vitro*	Model	References
PI3K/Akt/mTOR	Jiedu Tongluo Baoshen formula	*Radix Astragali, Ginseng, Cornus officinalis* Sieb. et Zucc*, Radix et Rhizoma Rhei, Dioscorea septemloba (Diocoreacea), Euryale ferox, Pyrrosiae Folium, Radix Salvia Miltiorrhizae, Hirudo*	*In Vivo*	rat model induced by high-fat diet and STZ	(8)
PI3K/Akt/mTOR	Dendrobium mixture	*Dendrobium, Radix Astragali, Schisandra, Radix puerariae, Salvia miltiorrhiza, Rehmanniae* and *Rhizoma anemarrhenae.*	*In Vivo*	STZ-induced rats	(9)
PI3K/Akt, AMPK	Yiqi Jiedu Huayu Decoction	*Radix Rehmanniae, Radix puerariae, S. M. Almeida ex Sanjappa and Predeep, Coptis chinensis, Franch, and Morus alba L*	*In Vivo*	STZ-induced rats	(10)
mTOR	Tongluo Digui decoction	*Hirudo, Carapax et Plastrum Testudinis, Radix Rehmanniae, Radix Astragali, Radix Angelicae Sinensi, Rhizoma Alismatis, Radix et Rhizoma Rhei*, and *Radix Glycyrrhizae*	*In Vivo*	STZ-induced rats	(11)
P62/LC3	Keluoxin capsule	*Radix Astragali, Radix Pseudostellariae, Lycium barbarum, Ligustrum lucidum, Hirudo, Radix et Rhizoma Rhei*	*In Vivo*	STZ-induced rats	(12)
p-AMPK/p-ULK1, mTOR/P70S6K, SIRT1	Tangshen Decoction	*Radix Astragali, Radix Pseudostellariae, Schisandra, Radix Rehmanniae, Chinese yam, Cornus officinalis Sieb.* et Zucc*, Poria cocos, Paeonia suffruticosa* Andr.*, Paeoniae Radix Rubra, Panax notoginseng*	*In Vivo*	STZ-induced rats	(13–15)
AMPK/mTOR, SIRT1	QiDiTangShen granules	*Radix Astragali, Rehmanniae radix praeparata, Euryale Semen, Sophorae Tonkinensis Radix et Rhizoma, Hirudo, Radix et Rhizoma Rhei, Baihuasheshecao*	*In Vivo*	db/db mice	(16)
mTORC1	Tangshenning formula	*Radix Astragali, Rhei Radix Et Rhizoma, Chuanxiong Rhizoma*, and *Rosae Laevigatae Fructus*	*In Vitro*	podocyte induced by HG	(17)
SIRT1/NF-κB	Yishen capsules	*Radix Astragali, Angelica sinensis, Euryale ferox, Alisma orientalis*, and *Rhodiola rosea*	*In Vivo/In Vitro*	STZ-induced rats, mouse podocytes	(18)
mTOR/PINK1/Parkin	Astragalus mongholicus Bunge and Panax notoginseng (Burkill) F.H. Chen formula	*Radix Astragali, Panax notoginseng, Radix Angelicae Sinensi, Achyranthes bidentata Blume*, and *Ecklonia kurome Okamura*	*In Vivo/In Vitro*	STZ-induced rats, mouse renal mesangial cells induced by HG	(19)
P62/LC3	Qidan Dihuang decoction	*Radix Astragali, Radix Salvia Miltiorrhizae, Radix Rehmanniae, Chinese yam*, and *Liquorice*	*In Vivo/In Vitro*	db/db mice and HG-cultured rat renal tubular epithelial cells	(20)
PLZF	Tangshen formula	*Radix Astragali, Euonymus alatus, Radix Rehmanniae, Citrus aurantium*L.*, Cornus officinalis* Sieb. et Zucc*, Radix et Rhizoma Rhei, Panax notoginseng*	*In Vivo/In Vitro*	db/db mice, HG-induced rat renal tubular epithelial cells	(21)

**Table 2:** The role of single herbs against renal injuries *via* regulating autophagy in DKD.

Signaling Pathways	Single herbs	*In Vivo/In Vitro*	Model	References
LC3-II-RIP-p38MAPK	*Cassia auriculata leaf extract*	*In Vivo/In Vitro*	STZ-induced rats, rat glomerular endothelial cells induced by HG	(22)
PI3K/Akt/mTOR	Radix astragali	*In Vivo/In Vitro*	STZ-induced mice, mouse podocytes	(23)

**Table 3:** The role of active compounds against renal injuries *via* regulating autophagy in DKD.

Signaling Pathways	Herbal extracts	Categories	Resource	*In Vivo/In Vitro*	Model	References
AMPK/mTOR	Dencichine	Others	*Panax notoginseng*	*In Vivo*	STZ-induced rats	(24)
AMPK/mTOR	Dioscin	Glycosides	the rhizome of Dioscorea nipponica Makino, D. *zingiberensis* C. H. Wright, D. *futschauensis* Uline	*In Vivo*	STZ-induced rats	(25)
mTOR/ULK1	Resveratrol	Polyphenols	Polygonum cuspidatum, mulberries and raspberries	*In Vivo*	STZ-induced rats	(26)
PI3K/Akt	Celastrol	Terpenoids	*Tripterygium wilfordii* Hook F	*In Vivo*	STZ-induced rats	(27)
AGE-RAGE-Nox4 axis	Salvianolic acid A	Phenolic acid	*Salvia miltiorrhiza*	*In Vivo*	STZ-induced rats	(28)
CaMKK2/AMPK/mTOR, PINK1/Parkin	Jujuboside A	Terpenoids	*Semen Ziziphi Spinosae*	*In Vivo*	STZ-induced rats	(29)
Nrf2 and PINK1	Astragaloside II	Glycosides	*Radix Astragali*	*In Vivo*	STZ-induced rats	(30)
P62/LC3	*Cordyceps militaris* polysaccharides	Others	*Cordyceps militaris*	*In Vivo*	STZ-induced mice	(31)
Bcl-2	Wogonin	Flavonoid	*Scutellaria baicalensis*	*In Vivo/In Vitro*	STZ-induced mice, mouse podocyte induced by HG	(32)
GSK3β	Sarsasapogenin	Glycosides	Anemarrhena asphodeloides Bunge	*In Vivo*	STZ-induced rats, mouse podocyte induced by HG	(33)
HMOX-1/Sirt1/AMPK	Puerarin	Flavonoid	*radix puerariae*	*In Vivo/In Vitro*	STZ-induced mice, mouse podocyte induced by HG	(34)
Pim1-p21-mTOR axis	Hispidulin	Flavonoid	*Salvia involucrata, Crossostephium Chinese, Arrabidaea chica, Grindelia argentina, Arrabidaea chica*, and *Saussurea involucrate*	*In Vitro*	mouse podocyte induced by HG	(35)
mTOR/TFEB	Catalpol	Glycosides	*Radix Rehmanniae*	*In Vivo/In Vitro*	STZ-induced mice, mouse podocyte induced by HG	(36)
mTOR/Twist1	Tripterygium glycoside	Glycosides	*Tripterygium wilfordii* Hook F	*In Vitro*	mouse serum-induced podocytes	(37)
β-arrestin-1	Tripterygium glycoside	Glycosides	*Tripterygium wilfordii* Hook F	*In Vitro*	mouse serum-induced podocytes	(38)
PI3k/Akt/mTOR	Curcumin	Others	*Curcuma longa*	*In Vivo/In Vitro*	STZ-induced rats, mouse podocyte	(39)
AKT/GSK3 β/β-Catenin	Ginsenoside Rg1	Glycosides	*Ginseng*	*In Vivo/In Vitro*	STZ-induced mice, mouse podocyte induced by HG	(40)
mTOR/P70S6K/4EBP1	Berberine	Alkaloids	Berberis vulgaris, Coptis Chinensis, and Berberis aristate	*In Vitro*	HG-cultured mouse podocytes	(41)
SIRT-NF-κB p65 axis	Astragaloside IV	Glycosides	*Radix Astragali*	*In Vivo/In Vitro*	KK-Ay mice, HG-cultured mouse podocytes	(42)
AMPK	Astragaloside IV	Glycosides	*Radix Astragali*	*In Vivo/In Vitro*	STZ-induced mice, HG-cultured mouse podocytes	(43)
miR-141-3p/PTEN/Akt/mTOR	Triptolide	Terpenoids	*Tripterygium wilfordii* Hook F	*In Vivo/In Vitro*	STZ-induced rats; HG induced human mesangial cells	(44)
miR-5189-5p/AMPK	Trigonelline	*Alkaloids*	*Trigonella foenum-graecum*	*In Vitro*	HG-cultured human mesangial cell	(45)
RAGE/mTOR	Paeoniflorin	Glycosides	*Paeonia lactiflora* Pall.	*In Vitro*	AGEs-induced mesangial cells	(46)
PI3k/Akt/ NF-κB	Wogonin	Flavonoid	*Scutellaria baicalensis*	*In Vivo/In Vitro*	STZ-induced rats, HG-cultured human renal tubular epithelial cells	(47)
PI3k/Akt	Curcumin	Others	*Curcuma longa*	*In Vitro*	AGEs-cultured rat renal tubular epithelial	(48)
PI3k/Akt	Astilbin	Flavonoid	*Smilax glabra rhizomes, Hypericum perforatum*	*In Vitro*	HG induced human renal tubular epithelial cells	(49)
miR-192-5p/GLP-1R	Icariin	Flavonoid	*Epimedium*	*In Vivo/In Vitro*	STZ-induced rats, HG-cultured human renal tubular epithelial cells and rat renal fibroblasts	(50)
NOX4	Complanatoside A	Glycosides	*Semen Astragali Complanati*	*In Vivo/In Vitro*	high-fat diet/STZ-induced diabetic model, TGF-β1-induced HK-2 cells	(51)
AMPK/mTOR	Cyclocarya paliurus triterpenic acids fraction	Terpenoids	*Cyclocarya paliurus*	*In Vivo/In Vitro*	STZ-induced rats, HG-induced HK-2 cells	(52)
TGF-βR1	Asiatic acid	Terpenoids	*Cyclocarya paliurus*	*In Vivo/In Vitro*	STZ-induced rats, HG+TGF-β1-induced HK-2 cells	(53)

## 2 Autophagy

Autophagy is a highly conserved lysosomal degradation pathway that ensures homeostasis by breaking down misfolded proteins, damaged organelles, and invasive pathogens for clearance and reuse (54). The three classical types of autophagy include macroautophagy, microautophagy, or chaperone mediated autophagy (55). Macroautophagy refers to the formation of an autophagosome with bilayer vesicles that envelops intracellular substances and eventually fuses with lysosomes to form an autophagosome, resulting in the degradation of the contents (56). In general, autophagy refers to macroautophagy. Microautophagy is a process in which lysosome or vacuole membrane directly invaginates cytoplasmic substances or organelles for degradation without the formation of autophagosomes (57). The molecular chaperone protein Hsc70 specifically recognizes the substrate protein molecule with the KFERQ group, binds to it, and then transports to the lysosome *via* the receptor LAMP2A on the lysosome membrane, which is finally degraded by lysosome hydrolase (58). According to the selectivity of its degradation substrates, autophagy can be divided into non-selective autophagy and selective autophagy (59). Non-selective autophagy degradation substrates are relatively non-selective, such as rapamycin induced autophagy, nitrogen source starvation induced autophagy, serum starvation induced autophagy, etc (60). Selective autophagy refers to the direct binding of selective autophagy receptors with LC3 (Atg8 in yeast and plant cells) to deliver specific degradation substrates to autophagosomes for degradation (61). The selective autophagy types found so far include mitochondrial autophagy, ribosomal autophagy, endoplasmic reticulum autophagy, peroxisome autophagy, etc (62).

Autophagy occurs through a series of successive stages, including autophagic initiation, nucleation, elongation, fusion and degradation, which are highly regulated by ATG proteins and other cofactors (63, 64). In the initiation stage of autophagy, the single or double layer membrane structure is formed in the cytoplasm and continuously expanded, which is phagophore (65). In the mature stage, phagophore continues to extend and completely envelops autophagy substances to form autophagosomes (66). In the fusion stage, the autophagosome and lysosome fuse to form autophagolysosome. The inner membrane of the autophagosome is degraded by lysosome enzyme, and the cargos in the autophagosome are degraded by lysosome enzyme. Small molecular substances such as amino acids and fatty acids produced are returned to the cytoplasm for reuse to maintain cell homeostasis (67). Thus, during autophagy, the formation, maturation, and subcellular reorientation of autophagosomes eventually lead to their fusion with lysosomes. The activity of the autophagy pathway can be demonstrated by the relative rate of autophagy substrate turnover, known as autophagy flux (68).

Researchers have identified molecular mechanisms involved in autophagy in yeast. Autophagy, as a common physiological regulatory mechanism in mammalian cells, has received extensive attention in recent years. The mammalian target protein of rapamycin and AMP kinase are two key molecules that regulate autophagy activity (69, 70). mTOR, a serine/threonine protein kinase, is a major component of the mTOR pathway and can form mTORC1 and mTORC2 complexes with other proteins (71). mTORC1 complex is a key regulator that controls growth and metabolism by integrating growth factors with nutrient signals such as amino acids, glucose and energy status (72). As a nutrient and energy sensor, AMPK can sense the level of ATP in cells and regulate cell metabolism and energy homeostasis (73). mTOR generally inhibits initiation of autophagy, which is regulated by the upstream regulator AMPK. In the presence of nutrient deficiency, AMPK is activated by high AMP levels, thereby increasing the function of tuberous sclerosis Complex 1(TSC1) and TSC2 and inhibiting the activity of GTP-binding proteins Rheb and mTOR (74). The inactivation of mTOR activates the UNC-51 like autophagy activating kinase 1 (ULK1) by dephosphorylation, the ULK1 complex (ULK1/2, ATGl3, ATG101, and FIP200) is then activated to initiate autophagy (75).

Autophagy extension is the continuous extension process of autophagy membrane with the participation of autophagy proteins (76). In this process, two ubiquitin-like binding systems of autophagy play an important role: the ATG5-ATG12-ATG16L ubiquitin-like binding system and the microtubule-associated protein 1 light chain 3(LC3) ubiquitin-like binding system. Ubiquitin E1-like ligase ATG7 non-covalently binds to ATG12 to form the ATG12-ATG7 complex, which, under the action of the E2-like enzyme ATG10, transfers ATG12 to ATG10 and then ATG12 to ATG5 (77). The two are covalently combined to form a complex, which can be combined with ATG16L to form ATG12-ATG5-ATG16L ternary complex. LC3 is also a ubiquitin-like protein with two forms, LC3-I and LC3-II (78). The lipoification of LC3-I to LC3-II is catalyzed by enzymes like E1, E2 and E3 (79). Under the catalysis of E1-like enzyme ATG7, the ATG7-LC3 complex is formed, and LC3 is transferred to E2-like enzyme ATG3 (80). Under the action of E3-like enzyme ATG12-ATG5-ATG16L, the phosphatidyl ethanolamine is added to the glycine residues at the carboxyl terminal of LC3-I to form LC3-II. LC3-PE can be stably bonded to the bilayer membrane of the autophagic vesicle. The two connective systems cooperate to promote the extension of autophagosome membrane (81). LC3-I is usually free in the cytoplasm, but once transformed into LC3-II, it will bind specifically to the membrane of autophagy and act as a marker protein for detecting autophagy. Western blot detection of LC3-II and LC3-I and observation of green fluorescence after transfection of GFP-LC3-II plasmid is a classical way to determine whether autophagy occurs in cells (82). In the fusion stage of autophagosome and lysosome, LC3-II in mature autophagosome is degraded by lysosome activity or converted into LC3-I by Atg4B in the outer membrane (83).

## 3 Autophagy in diabetic kidney disease

Autophagy homeostasis is essential for normal kidney function, and abnormal renal autophagy is closely related to the occurrence of DKD (84, 85). Abnormal autophagy has been reported to play a role in renal fibrosis and inflammation in DKD (38, 86). Autophagy dysfunction leads to the accumulation of damaged organelles, such as mitochondria, which play a major role in the formation of reactive oxygen species (ROS) (87, 88). This leads to functional impairment of various renal intrinsic cells, such as tubular epithelial cells, podocytes, and endothelial cells. In short, multiple diabetes risk factors lead to damage to every parenchymal cell type that makes up the kidney. Restoration of autophagy can improve renal function, inhibit renal inflammation and fibrosis, delay the progression of DKD, and become an important target for the treatment of DKD.

### 3.1 Podocytes and autophagy

The basic level of autophagy of podocytes is significantly higher than that of other innate cells of kidney, and such high level of autophagy is necessary to maintain the normal physiological function of podocytes (89). It was found that the abnormal autophagy pathway of podocyte is closely related to the pathogenesis of DKD. Animal studies showed that autophagy substrate protein p62 accumulated in DKD model podocytes, indicating that autophagy activity of kidney podocytes was inhibited under DKD condition (90, 91). Podocytes are the intrinsic cells of the kidney. The podocytes issued by the adjacent podocytes are wrapped around the basement membrane of the glomeruli, and form a hiatus diaphragm complex through interlacing adhesion molecules and proteoglycan molecules to maintain the morphology and structure of the basement membrane of the glomeruli, regulate the charge barrier to prevent the passage of large molecular proteins, stabilize the structure of the capillaries of the glomeruli, balance the static pressure of the capillaries, and maintain the function of the glomerular endothelial cells It can synthesize and secrete vascular endothelial growth factor.

Compared with diabetic renal biopsy samples with mild urinary protein excretion, renal samples from diabetic patients had less pathological damage and significantly decreased podocyte autophagy level. Clinical and animal experiments showed that autophagy was impaired in type 2 diabetic patients and OLETF rats with abundant albuminuria (92). Renal mesangial dilatation and glomerulosclerosis were more severe after specific ATG5 knockout in STZ-induced diabetic mouse podocytes, suggesting a critical role of podocyte autophagy in glomerulopathy (93). In addition, when podocyte-specific ATG5 knockout mice were given a high-fat diet, they developed abundant albuminuria, accompanied by severe tubulointerstitial lesions and podocytic damage, resulting in abnormal lysosome accumulation, compared with wild-type mice (93). Interestingly, autophagy levels of podocytes in DKD varied with exposure time. *In vitro* experiments showed that autophagy level increased in mouse podocytes lines induced by short-term high glucose. Long-term exposure to high sugar levels inhibited autophagy (94). In streptozotocin (STZ) -induced diabetic mice, podocyte autophagy was induced at 4 weeks and inhibited at 8 weeks (95).

### 3.2 Glomerular mesangial cells and autophagy

Glomerular mesangial cells (GMCs) are one of the most important intrinsic cells in glomeruli and play a crucial role in the occurrence and development of glomerular diseases (96, 97). Any intervention that maintains the balance between mesangial cell proliferation, hypertrophy, and apoptosis, and improves mesangial stroma metabolism, is key to alleviating or reversing glomerular disease. The renal pathological changes of DKD are mainly characterized by proliferation of mesangial cells and increased mesangial matrix. No matter mesangial cell proliferation or atrophy, mesangial extracellular proteins are associated accumulation, resulting in sclerosis of the glomeruli.

Studies have shown that mesangial autophagy is induced by transforming growth factor β1(TGF-β1) through TAK11 and PI3K-Akt dependent pathways in serum-free conditions, which prevents mesangial cell apoptosis and increases mesangial cell survival (98). Autophagy can negatively regulate mesangial cells to produce extracellular matrix through intracellular degradation of type I collagen (99). This suggests a new intracellular mechanism, mesangial cells reduce the level of collagen fibers through autophagy, thus inhibiting renal fibrosis, which is the main mechanism of the occurrence and development of CKD and DKD (100).

*In vitro* experiments also showed changes in autophagy of GMCs at DKD stage. The expression of metalloproteinase inhibitor 3 gene (Timp3) was down-regulated in the kidneys of diabetic patients, in the kidneys of STZ-induced diabetic mice, and in GMCs treated with high glucose (101). Studies using Timp3^−/−^ mice and high-sugar-induced GMCs have shown that Timp3 gene deletion inhibits autophagy and exacerbates diabetic kidney injury in mice by reducing the expression of FOXO1a and its downstream autophagy related genes. After high expression of Timp3 by adenovirus, the autophagy disorder of GMCs restored by Timp3 gene was reversed and the kidney injury was improved. Mitochondrial autophagy levels are decreased in age-induced GMCs, and the recovery of autophagy significantly reverses mitochondrial dysfunction and apoptosis (102). Despite the above proof of the protective effect of autophagy on GMCs damage, its specific mechanism still needs to be further studied and verified.

### 3.3 Proximal renal tubular epithelial cells and autophagy

Proximal renal tubular epithelial cells (PTEC) are the most abundant cell type in the kidney, which is essential for normal kidney function and organ regeneration after injury. Proximal renal tubules are a key site of reabsorption activity in the kidney, making it a highly metabolically active organ, second only to the brain in terms of oxygen consumption. Evidence from recent studies suggests that PTECs play a leading role in nephron damage and renal dysfunction during diabetes, which is referred to as “diabetic tubular disease” (103). Due to their position in the nephron and major reabsorption, PTECs are often exposed to a variety of toxic factors in glomerular filtrate, such as high glucose, albumin, and advanced glycation end products (AGEs), which are associated with progressive interstitial inflammation and fibrosis (104, 105).

In physiological state, renal PTEC is in autophagic homeostasis. However, active transport of tubular epithelial cells consumes a lot of energy, making these cells more susceptible to hypoxia or energy deprivation (106). In addition, the renal medulla provides a lower oxygen environment than the renal cortex, which is more likely to lead to tubular epithelial cell damage (107). Autophagy ensures the normal function of cells in harsh environments, which is particularly important for tubular epithelial cell. When autophagy disorders occur in PTEC, structural changes will occur in the kidney, such as interstitial fibrosis (107, 108). In particular, PTEC autophagy disorder can cause mitochondrial damage and lead to severe renal homeostasis abnormalities (109, 110). Although the autophagy level of renal PTECs is relatively low under physiological conditions. However, selective Atg5 or Atg7 deletion in the proximal tubules in mice resulted in progressive renal injury and senescence, characterized by mitochondrial damage, accumulation of ubiquitinated aggregates, increased apoptosis, and tubulointerstitial fibrosis (111, 112). In contrast, the kidneys of mice with ATG5-deficient distal tubules or collecting tubules showed no significant histopathologic changes (113, 114). These results suggest that, under normal circumstances, low levels of basal autophagy are essential for maintaining PTEC function and that proximal tubules are more dependent on basal autophagy than other tubule segments.

Previous studies have shown autophagy disorders in kidney biopsy samples from DKD patients, diabetic animal models, and high-glucose treated PTECs. Mechanism studies have demonstrated that p53 is activated in DKD, thereby inducing the expression of miR-214 in renal tubules (115, 116). miR-214 directly targets ULK1, impedes initiation of autophagy, and leads to impaired autophagy of PTECs. More and more studies have shown mTOR activity dependent autophagy disorders in diabetic animal models and PTEC cells induced by high glucose or albumin overload (111, 117). Long-term or excessive albumin in glomerular filtrate of diabetic nephropathy patients can eventually impair autophagy of PTECs through mTOR dependent mechanism, leading to tubular injury. Studies have shown that obesity-induced autophagy inhibition in PTECs in type 2 diabetes patients and mice is associated with mTOR activation, and that the mTOR inhibitor rapamycin significantly improves renal autophagy deficiency in obese mice induced by a high fat diet (118). Long-term albumin overload resulted in decreased expression of BNIP3L, and then mitochondrial autophagy disorder occurred in PTECs (119, 120). mTOR activity in PTECs and SIRT1 pathway can be inhibited and autophagy disorder can be significantly reversed in diabetic Wistar fat rats after low protein diet and 40% daily food intake restriction, thus improving diabetic renal tubular injury (121, 122).

## 4 Chinese herbal medicine and its active compounds in attenuating renal injury *via* regulating autophagy in diabetic nephropathy

### 4.1 Chinese herb compound preparations in attenuating renal injury *via* regulating autophagy

Lately, Chinese herbal medicine and its active compounds are increasingly considered as alternative sources for modulating renal autophagy in DKD. Jiedu Tongluo Baoshen formula (JTBF), a mixture of nine crude drugs, reduced early clinical proteinuria in the treatment of DKD patients (123). In high-fat diet and streptozocin (STZ) induced rats, Jin et al. found that JTBF activated the expression of podocyte autophagy-related proteins (beclin-1, LC3 and P62) *via* suppressing PI3K/Akt/mTOR (8). Furthermore, they found that the extracts of JTBF included 77 active compounds by high performance liquid chromatography (HPLC) (8). Dendrobium mixture is a Chinese herb compound preparation for treating diabetes and its complications, which lowers glucose and lipid levels, and alleviates insulin resistance (124). In STZ-induced rats, Dendrobium mixture has protective effects on the kidney through suppressing the PI3K/Akt/mTOR signaling pathway and promoting renal autophagy (9). In addition, Yiqi Jiedu Huayu decoction also regulated the PI3K/Akt and AMPK pathways, thus promoted autophagy in STZ-induced rats (10). The changes in these key molecules suggest that PI3K/Akt/mTOR signaling pathway is the prototypical signaling pathway that regulates autophagy, and mTOR is a key target of autophagy regulation and plays a negative role in regulating autophagy.

Keluoxin capsule is a Chinese herb compound preparation for treatment of qi and yin deficiency and blood stasis in DKD (125). A meta-analysis for DKD found that Keluoxin capsule improved the levels of serum creatinine and lipid (126). A STZ-induced rat model was used by some researchers to demonstrate the protective effects of Keluoxin capsule

*in vivo*, they found Keluoxin capsule regulated autophagy in podocytes *via* increasing the expression of LC3-II and p62 proteins (12).

Tangshen Decoction, so-called Tangshenjian, is a Chinese herb compound preparation for DKD that effectively improve glucose and lipid metabolism and reduce urine protein (127, 128). Unc-51-like kinase 1 (ULK1) is an autophagy-related protein, which regulates by AMPK (129). A study proved that Tangshen Decoction significantly enhanced podocyte autophagy through modulation of p-AMPK/p-ULK1 signaling in STZ-induced rats (13). Ribosomal protein S6 kinase beta-1 (P70S6K) is one of mTOR downstream factors. Meanwhile, these researchers also confirmed that Tangshen Decoction regulated podocyte autophagy by mTOR/P70S6K pathway (14) and up-regulated renal tubule autophagy by regulating SIRT1 (15).

QiDiTangShen granules, a Chinese herb compound preparation, has been proven to effectively reduce proteinuria of DKD (130). Studies have reported that QiDiTangShen granules alleviated renal injuries *via* modulating the gut microbiome composition and improving bile acid profiles in DKD (131). Recent studies have reported that QiDiTangShen granules also activated autophagy through the regulation of nutrient-sensing signal pathways in db/db mice (16).

Tongluo Digui decoction is a Chinese herb compound preparation that has been used for decades in the treatment of DKD, which improved the clinical symptoms and renal function of patients with DKD in stage IV, reduced proteinuria, relieved inflammatory state and improved renal tubular injury (132, 133). *In vivo* experiments have demonstrated that Tongluo Digui decoction protected podocytes and reduced proteinuria through increasing autophagy by inhibiting mTOR phosphorylation in STZ-induced rats (11). Furthermore, another study reported that it alleviated renal inflammation in diabetic mice by reduce the expression of NLRP3 (134).

#### 4.1.1 Chinese herb compound preparations in regulating autophagy of podocyte

Tangshenning formula (TSN) is a Chinese herb compound preparation to reduce proteinuria and improve renal function in patients with DKD (135). Some scholars have confirmed through *in vivo* and *in vitro* experiments that TSN ameliorated podocyte epithelial-mesenchymal transformation *via* inhibiting the Wnt/β-catenin pathway in KK-Ay mice (136), and restored podocyte autophagy *via* inhibiting mTORC1 pathway in HG-induced podocyte (17).

Yishen capsule is a Chinese herb compound preparation that has been used for decades in the First Affiliated Hospital of Shanxi Medical University (137, 138). In previous studies, it has been found that Yishen capsule restored podocyte foot process effacement (137) and alleviated renal inflammation by regulating NOD-like receptor signaling pathway in DKD mice (139). A recent study has found that, Yishen capsule improved renal injury by promoting podocyte autophagy *via* the SIRT1/NF-κB pathway in STZ-induced DKD rats (18). Network pharmacology and molecular docking found that Yishen capsule included in 12 key components (beta-sitosterol, beta-carotene, stigmasterol, alisol B, mairin, quercetin, caffeic acid, 1-monolinolein, kaempferol, jaranol, formononetin, and calycosin) (140), and reduced renal inflammation and fibrosis damage *via* upregulating the expression HIF-1α and inhibiting JAK/STAT signaling pathways (141).

#### 4.1.2 Chinese herb compound preparations in regulating autophagy of glomerular mesangial cells and proximal renal tubular epithelial cells

Astragalus mongholicus Bunge and Panax notoginseng (Burkill) F.H. Chen formula (APF) is a Chinese herb compound preparation to significantly alleviate renal inflammation (142). Phosphatase and tensin homolog-induced kinase 1 (PINK1)/Parkin pathway is an important link in the regulation of mitophagy (143). A study demonstrated that APF also alleviated renal inflammation by upregulating autophagy of glomerular mesangial cells *via* suppressing mTOR and activating PINK1/Parkin signaling (19).

Qidan Dihuang decoction (QDD) is a Chinese herb compound preparation developed according to the etiology and pathogenesis of DKD in TCM, namely “deficiency of Qi and Yin, blood stasis”. QDD is composed of Radix Astragali, Radix Salvia Miltiorrhizae, Radix Rehmanniae, Chinese yam, and Liquorice, which inhibits renal fibrosis *via* modulating RAS system in STZ-induced rats (144). In db/db mice and HG-cultured rat renal tubular epithelial cells (NRK-52E), Liang et al. further found that QDD promoted renal autophagy by downregulation of the ratio of LC3-II/LC3-I and upregulation of P62 (20).

Tangshen Formula (TSF), a Chinese herb compound preparation, significantly extenuated proteinuria and improved the estimated glomerular filtration rate (eGFR) among DKD patients in a multicenter double-blind randomized placebo-controlled trial (145). TSF had six components in rat plasma, including astragaloside IV, notoginsenoside R1, ginsenoside Re, ginsenoside Rb1, loganin, and morroniside (146). Promyelocytic Leukemia Zinc Finger (PLZF) was dispensable for ILC3 development (147). Some researchers have confirmed through *in vivo* and *in vitro* experiments that TSF attenuated diabetic renal injuries by upregulating autophagy *via* inhibition of PLZF expression (21).

### 4.2 Single herbs in attenuating renal injury *via* regulating autophagy

Radix astragali, a medicinal material for tonifying Chinese Qi that is widely used in China to treat kidney diseases. Recent study found that Radix astragali enhanced podocytes autophagy and delays DKD probably by inhibiting the PI3K/Akt/mTOR pathway (23). In addition, Cassia auriculata leaf extract ameliorates renal injury by attenuating autophagic necroptosis *via* receptor-interacting protein kinase (RIP)-1/RIP-3-p-p38MAPK signaling in STZ-induced rats and rat glomerular endothelial cells induced by HG (22).

### 4.3 The active compounds in attenuating renal injury *via* regulating autophagy

It is known that the active compounds can attenuate renal injury by regulating autophagy. Dencichine is a non-protein amino acid from *Panax notoginseng (* 148) with a wide range of biological activities. Recent studies found that Dencichine ameliorated renal injury by increasing autophagy through regulating AMPK/mTOR pathway (24). Dioscin is a glycoside of steroidal saponin, which is found in the rhizome of Dioscorea nipponica Makino, D. *zingiberensis* C. H. Wright, D. *futschauensis* Uline and other plants (149). Previous research studies reported that Dioscin has a potent effect against hyperuricemic (150, 151), ischemia/reperfusion injury (IRI) (152), inflammation (153–157), oxidative stress (154, 156, 158), and fibrosis (156). Zhong et al. found Dioscin enhanced autophagy *via* an AMPK/mTOR pathway in STZ-induced DKD rats (25).

Resveratrol is a natural non-flavonoid phenol, mainly found in Polygonum cuspidatum, mulberries and raspberries (159). Emerging evidence suggests that Resveratrol decreased mTOR/ULK1-mediated autophagy in nephrons in STZ-induced rats (26). Celastrol is a triterpene of *Tripterygium wilfordii* Hook F, which contributes to renal protection (160). Studies have proved that Celastrol also achieved podocyte homeostasis by regulating autophagy *via* PI3K/Akt pathway (27).

Salvianolic acid A is a water-soluble phenolic acid extracted from *Salvia miltiorrhiza*, their various pharmacological effects such as anti-inflammation and anti-diabetic (161, 162) have been the focus of research for many years. A study reported that Salvianolic acid A restored the disturbed autophagy in glomerular endothelial cell *via* AGE-RAGE-Nox4 axis (28). Jujuboside A, a triterpene saponin from *Semen Ziziphi Spinosae*, has several biological activities including anti-oxidant, anti-inflammation and anti-apoptosis (163). Another study also found that Jujuboside A enhanced autophagy *via* regulating calcium/calmodulin-dependent protein kinase kinase 2 (CaMKK2) -AMPK-mTOR and PINK1/Parkin pathways in DKD rats (29). *Radix Astragali* is one of the most widely used ‘‘benefiting Qi” herbs, which has renal protective effects on DKD (164). As one of the active constituents of *Radix Astragali*, Astragaloside II improved podocyte autophagy by regulating nuclear factor erythroid 2-related factor 2 (Nrf2) and PINK1 pathway (30).

*Cordyceps militaris* is an entomogenous fungus belonging to Cordyceps genus of Cordyceps family for the treatment of chronic kidney diseases (165). *Cordyceps militaris* polysaccharides is one of the most abundant constituents of *Cordyceps militaris*. Studies have found that *Cordyceps militaris* exhibits a variety of various pharmacological effects, such as antioxidant, anti-inflammatory, immunomodulatory, and antihyperlipidemic (166). Furthermore, another study found that *Cordyceps militaris* polysaccharides significantly increased the rate of renal autophagy through regulating the expression of p62 and LC3 (31).

#### 4.3.1 The active compounds in attenuating renal injury *via* regulating autophagy of podocyte

Wogonin, a flavonoid derived from the root of *Scutellaria baicalensis*, which has the pharmacological effects of antivirus, antitumor, anti-inflammation (167). In STZ-induced diabetic mice and HG induced mouse podocyte clone 5 (MPC5) cells, Wogonin can regulate the expression of Bcl-2 to promote autophagy and inhibit apoptosis in a dose-dependent manner (32). In addition, Wogonin mitigated NF-κB p65-mediated renal inflammatory response in STZ-induced diabetic mice (32). Sarsasapogenin, the main active ingredient in Anemarrhena asphodeloides Bunge, restored podocyte autophagy through targeting glycogen synthase kinase-3 β (GSK-3β) signaling pathway (33).

Radix puerariae is a major herbal medicine to treat patients with DKD in China. Puerarin, an active compound of Radix puerariae exerted renoprotective effects in STZ-induced mice with endothelial nitric oxide synthase (eNOS) deficiency (168). The evidence from a study suggests that Puerarin also protected podocytes through heme oxygenase 1 (HMOX1) and Sirt1-mediated upregulation of autophagy in DKD (34). Hispidulin is a natural flavonoid in a wide range of herbs, such as *Grindelia argentina, Arrabidaea chica*, *Saussurea involucrate, Salvia involucrata, Crossostephium Chinese*, and *Arrabidaea chica (* 169, 170). Hispidulin displays a wide range of pharmacological activities, including antifungal, anti-inflammatory, and antioxidant (171). At the same time, Hispidulin induced podocyte autophagy *via* the regulation of RCC1-related protein (Pim1)-p21-mTOR signaling axis (35).

Catalpol is an iridoid glycoside compound extracted from *Radix Rehmanniae*, which has been shown to have antioxidant and anti-inflammatory effects (172, 173). Similarly, as reported, Catalpol improved podocyte autophagy by inhibiting mTOR activity and promoting transcription factor EB (TFEB) nuclear translocation in STZ-induced mice and mouse podocyte induced by HG (36). Tripterygium glycoside is a fat-soluble mixture extracted from *Tripterygium wilfordii* Hook F, which composed of diterpene lactone, alkaloid and triterpenoid (174). Tripterygium glycoside has excellent anti-inflammatory and antioxidant damage properties and is widely used in China to treat proteinuria in patients with DKD (175, 176). Similarly, Tripterygium glycoside also alleviated podocyte apoptosis by upregulating autophagy through the mTOR/Twist-related protein 1 (TWIST1) signaling pathway (176) and downregulation of β-arrestin-1 (38).

Curcumin, a yellow phytochemical produced by *Curcuma longa*, has anti-inflammatory and antioxidant properties (38, 177).A finding of a study suggests that Curcumin induces podocyte autophagy through the PI3k/Akt/mTOR pathway (39). Curcumin as reported also activated autophagy in advanced glycation or glycoxidation end-products (AGEs)-cultured human renal tubular epithelial by the activation of PI3K/Akt pathway (48). Ginsenoside Rg1 is an active component of ginseng, which has multiple effects, including anti-inflammation, antioxidation and anti-apoptosis (178). Ginsenoside Rg1 also alleviated podocyte injury by regulating Akt/GSK3 β/β-Catenin pathway by restoring autophagic activity (40). Berberine is a quaternary ammonium salt originating from isoquinoline alkaloids discovered in Berberis vulgaris, Coptis Chinensis, and Berberis aristate (179). In HG-cultured mouse podocytes, Berberine activated podocyte autophagy by inhibiting the mTOR/P70S6K/4EBP1 signaling pathway (41). Further, several lines of evidence suggest that Astragaloside IV promoted podocyte autophagy *via* the SIRT-NF-κB p65 axis (42) and upregulating the expression of AMPK (43).

#### 4.3.2 The active compounds in attenuating renal injury *via* regulating autophagy of glomerular mesangial cells and proximal renal tubular epithelial cells

Triptolide is a diterpene of *Tripterygium wilfordii* Hook F, which exerts immune suppression, anti-inflammatory and anti-cancer activities (180). Similarly, Triptolide restored autophagy to alleviate diabetic renal fibrosis through the miR-141-3p/PTEN/Akt/mTOR pathway (44). Trigonelline is a natural alkaloid from *Trigonella foenum-graecum (* 181). In HG-cultured human mesangial cell, Trigonelline activated autophagy by up-regulating miR-5189-5p expression and activating the AMPK pathway (45). Paeoniflorin is the main bioactive component of *Paeonia lactiflora* Pall., which has commendable immune regulation and anti-inflammatory effects (182). In addition, Paeoniflorin alleviated AGEs-induced mesangial cell dysfunction by inhibiting autophagy through inhibiting RAGE and upregulating the level of p-mTOR (46).

Wogonin also alleviated renal tubular epithelial injury by inhibiting PI3K/Akt/NF-κB signaling pathways (47). Astilbin is a flavonoid compound from *Smilax glabra rhizomes, Hypericum perforatum*, which possesses strong anti-inflammation, immunosuppression and antioxidation activities (183). It is important to note that Astilbin inhibited autophagy and apoptosis through the PI3K/Akt pathway in HG-induced human proximal tubular epithelial cells (49).

Icariin is a flavonoid glycoside extracted from the medicinal plant *Epimedium (* 184). Icariin exerts renal protection through multiple bioactivities such as anti-fibrosis (50, 185), anti-inflammatory (186, 187), and anti-oxidative stress (186). In STZ-induced DKD rats and in HG-incubated human renal tubular epithelial cells and rat renal fibroblasts, Icariin restored autophagy through the miR-192-5p/Glucagon-like peptide-1 receptor (GLP-1R) pathway (50). Complanatoside A, an active component from Semen Astragali Complanati, might be a potential therapeutic agent for DKD by suppressing autophagy *via* targeting NOX4 inhibition in a high-fat diet/streptozotocin-induced diabetic model and TGF-β1-induced HK-2 cells (51).

*Cyclocarya paliurus* is a common edible and medicinal plant in China, also known as “sweet tea tree” (188). *Cyclocarya paliurus* triterpenic acids fraction ameliorated renal injury through AMPK-mTOR-regulated autophagy pathway (52). In addition, a triterpenic-acid extract of *Cyclocarya paliurus* had been shown to reduce renal fibrosis in DKD (189). In STZ-induced rats and HG+TGF-β1-induced HK-2 cells, asiatic acid, a triterpenic-acid extract of *Cyclocarya paliurus* suppressed tubulointerstitial fibrosis by activating the autophagy-lysosome system *via* inhibiting TGF-β type I receptor (TGF-βRI) (53).

To sum up, the above Chinese herbal medicine research review is shown in **Tables 1**–**3**. These active compounds are classified into six categories based on their chemical structure-polyphenols, alkaloids, terpenes and terpenoids, flavonoid, glycosides and others in **Table 3**.

## 5 Conclusions and perspectives

With the development of modern society, there are more and more patients with DKD. Due to the complex etiology of DKD, a single treatment method targeting autophagy often does not work ideally, and Chinese herbal medicine has unique advantages in the treatment of DKD. First of all, since the mechanism of autophagy involves multiple pathways, it is sometimes difficult for drugs with a single target to take into account multiple pathways. Chinese herbal medicine can take into account multiple targets of autophagy in DKD at the same time through multi-target action. Secondly, Chinese herbal medicine and its active compounds have been proven to be safe. Many Chinese herbal compound preparations, including Yiqi Jiedu Huayu Decoction, Keluoxin capsule, and many others mentioned above, have been used in China for thousands of years. In consideration of objective factors such as the mixing of ingredients in Chinese herbal compound preparations, researchers in recent years have attempted to elucidate the mechanism of action of Chinese herbal compound preparations by isolating the active ingredients and conducting cellular or animal experiments (190, 191).

At this moment, there are many shortcomings in the research on regulation of DKD autophagy in Chinese herbal medicine. The research on the mechanism of regulating DKD autophagy in Chinese herbal medicine is still at the stage of observational studies and lacks in-depth mechanism exploration. This is partly due to the use of natural products in Chinese herbal medicine, which is characterized by confounding (192). Therefore, more standardized and larger scale clinical studies are necessary to facilitate wider acceptance of Chinese herbal medicine treatment in DKD patients.

## Author contributions

PLiu, HZ, and PLi: conceptualization. PLiu, WZ, YW, and GM: writing - original draft preparation. HZ and PLi: editing, and revising. PLiu, HZ, and PLi: supervision. All authors contributed to the article and approved the submitted version.
